# Acquisition of an oncogenic fusion protein serves as an initial driving mutation by inducing aneuploidy and overriding proliferative defects

**DOI:** 10.18632/oncotarget.11716

**Published:** 2016-08-30

**Authors:** Jacob M. Loupe, Patrick J. Miller, Benjamin P. Bonner, Elaine C. Maggi, Jyothi Vijayaraghavan, Jovanny Zabaleta, Christopher M. Taylor, Fern Tsien, Judy S. Crabtree, Andrew D. Hollenbach

**Affiliations:** ^1^ Department of Genetics, Louisiana State University Health Sciences Center, New Orleans, LA, USA; ^2^ Department of Pediatrics and Stanley S. Scott Cancer Center, Louisiana State University Health Sciences Center, New Orleans, LA, USA; ^3^ Department of Microbiology, Immunology, and Parasitology, Louisiana State University Health Sciences Center, New Orleans, LA, USA; ^4^ Center for Human Genetic Research, Massachusetts General Hospital, Richard B. Simches Research Center, Boston, MA, USA; ^5^ Tulane University, New Orleans, LA, USA

**Keywords:** aneuploidy, Pax3-FOXO1, myogenesis, phosphorylation, alveolar rhabdomyosarcoma

## Abstract

While many solid tumors are defined by the presence of a particular oncogene, the role that this oncogene plays in driving transformation through the acquisition of aneuploidy and overcoming growth arrest are often not known. Further, although aneuploidy is present in many solid tumors, it is not clear whether it is the cause or effect of malignant transformation. The childhood sarcoma, Alveolar Rhabdomyosarcoma (ARMS), is primarily defined by the t(2;13)(q35;q14) translocation, creating the PAX3-FOXO1 fusion protein. It is unclear what role PAX3-FOXO1 plays in the initial stages of tumor development through the acquisition and persistence of aneuploidy. In this study we demonstrate that PAX3-FOXO1 serves as a driver mutation to initiate a cascade of mRNA and miRNA changes that ultimately reprogram proliferating myoblasts to induce the formation of ARMS. We present evidence that cells containing PAX3-FOXO1 have changes in the expression of mRNA and miRNA essential for maintaining proper chromosome number and structure thereby promoting aneuploidy. Further, we demonstrate that the presence of PAX3-FOXO1 alters the expression of growth factor related mRNA and miRNA, thereby overriding aneuploid-dependent growth arrest. Finally, we present evidence that phosphorylation of PAX3-FOXO1 contributes to these changes. This is one of the first studies describing how an oncogene and post-translational modifications drive the development of a tumor through the acquisition and persistence of aneuploidy. This mechanism has implications for other solid tumors where large-scale genomics studies may elucidate how global alterations contribute to tumor phenotypes allowing the development of much needed multi-faceted tumor-specific therapeutic regimens.

## INTRODUCTION

Aneuploidy is common in solid tumors, with nearly 90% of such tumors being aneuploid [1]. Despite the prevalence of aneuploidy in these cancers, it has not been firmly established if aneuploidy is an early step that drives malignant transformation, results as a consequence of transformation, or what role, if any, oncogenes play in this process. Although many solid tumors are characterized by a specific oncogenic mutation, very few reports examined the role that the resulting oncoprotein plays in driving the initial stages of tumor development, possibly through the acquisition of aneuploidy and in overcoming aneuploidy-dependent growth arrest. In many of these reports, oncogene-induced aneuploidy results from the overexpression of a protein that is central to the chromosome segregation machinery or regulates the process of chromosome segregation [2–6]. Although one report demonstrates that an oncogenic transcription factor, *TLX1*, is sufficient to induce aneuploidy in T-cell progenitors, the process involves a limited mechanism in which specific genes important for maintaining mitotic checkpoint control are affected [7].

Upon the acquisition of aneuploidy, a cell's natural response is to attenuate proliferation. Previous work demonstrated that the induction of aneuploidy in diploid or near diploid cell lines resulted in significant proliferative defects [8, 9], raising an apparent contradiction for tumor progression: rapidly proliferating tumor cells are often aneuploid, a state that under normal circumstances is not conducive to proliferation [10]. Therefore, tumor cells must develop a mechanism to overcome aneuploid-dependent proliferative defects and develop an enhanced proliferation rate. However, the mechanism by which an oncogene promotes aneuploidy in pre-cancerous cells, how these cells overcome the adverse proliferative effects associated with this state, and whether post-translational modifications can regulate this process are important, yet unanswered, questions in the development of solid tumors.

Like other solid tumors, aneuploidy is common in Rhabdomyosarcoma (RMS) [11], which accounts for nearly half of childhood soft tissue sarcomas. RMS is comprised of two main subtypes: embryonal (ERMS) and alveolar (ARMS), each defined by its unique histology, clinical presentation, therapy, and prognosis [12]. ARMS, the more aggressive subtype, is primarily defined by the t(2;13)(q35;q14) translocation, which creates the oncogenic fusion protein PAX3-FOXO1 [13, 14]. In addition to the defining cytogenetic abnormality, ARMS tumor cells contain cytogenetic evidence of polyploidy, having cells ranging in chromosome number from hypodiploid to hypertetraploid, with a wide and heterogeneous range of chromosome number in cells within a single patient sample [11, 15–18]. There were also instances of chromosome amplification as double minutes and heterogeneously staining regions, gains and losses through insertions and deletions, derivative chromosomes and additional translocations, and breakpoints around the centromere [11, 15–18]. Despite extensive work understanding the altered molecular characteristics of PAX3-FOXO1 relative to PAX3 [19–25] and the knowledge that phosphorylation of the fusion protein contributes to ARMS tumor phenotypes [26], it is not known what role, if any, the fusion protein plays in the promotion of aneuploidy and chromosomal structural abnormalities, overcoming aneuploidy-dependent proliferative defects, and whether phosphorylation of the fusion protein contributes to this process.

In this study we examine how the expression of PAX3-FOXO1 affects global mRNA and miRNA expression. We are the first to show that the presence of PAX3-FOXO1 is sufficient to alter mRNA and miRNA expression, either through direct regulation of genes or indirectly through downstream effects, subsequently altering protein levels important for multiple aspects of chromosome number and structure, thereby promoting the aneuploid state. Further, we are the first to demonstrate that the presence of an oncogene is sufficient to alter, either directly or indirectly, the expression of multiple growth related mRNA and miRNA to overcome aneuploidy-induced proliferative defects. In addition, inhibition of phosphorylation at Ser201 or Ser205 on PAX3-FOXO1 reversed oncogene dependent mRNA and miRNA changes, and inhibited the proliferation of cells containing chromosomal abnormalities. Our results allow us to propose a model by which the expression and phosphorylation of an oncogene, PAX3-FOXO1, serves as the driving molecular event to initiate the development of a solid tumor by reprograming cells to induce aneuploidy and override aneuploidy-induced growth arrest.

## RESULTS

### PAX3-FOXO1 is sufficient to alter the expression of mRNA and miRNA to promote aneuploidy

**Figure 1:**
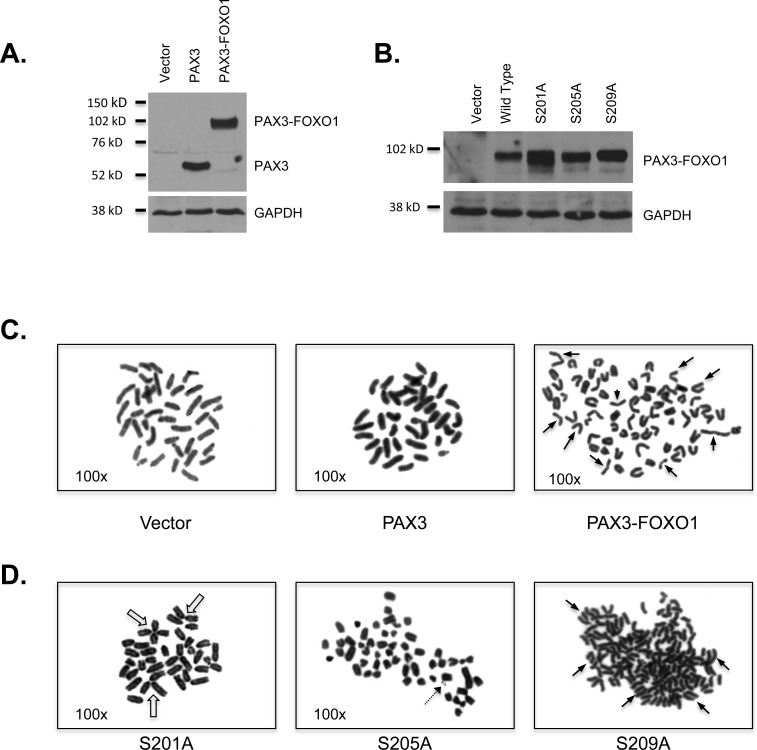
The expression of PAX3-FOXO1 promotes aneuploidy and chromosomal structural abnormalities Expression of **A.** PAX3, PAX3-FOXO1 or **B.** PAX3-FOXO1 phosphomutants. Total extracts were made from stably transduced cells and protein was determined using an antibody specific for PAX3, as described in the Materials and Methods. Representative picture of a metaphase chromosome analysis for **C.** cells stably transduced with empty vector, PAX3 or PAX3-FOXO1 or **D.** individual PAX3-FOXO1 phosphomutants. The closed arrows indicate representative sister chromatid dissociation, the open arrow indicate representative telomere association, and the dotted arrow indicate a representative double minute.

To understand how PAX3-FOXO1 affects global mRNA and microRNA (miRNA) expression, we stably transduced passage-matched mouse primary myoblasts with the MSCV-IRES-puromycin retroviral vector (negative control), or the same retroviral vector expressing FLAG-epitope tagged PAX3 (FLAG-PAX3), or FLAG-PAX3-FOXO1 (Figure 1A), a tag previously shown to not affect Pax3 or Pax3-FOXO1 function [24, 26]. The transduced cells were selected with puromycin; selected cells were harvested from three independent transductions and pooled, resulting in a single mixed population for each individual construct. By utilizing a population of transduced cells, we remove the potential for variability that may occur from clonal effects. The level of PAX3-FOXO1 expression we observed is equivalent to the level of expression of the fusion protein in ARMS tumor cell lines (Supplementary Figure 1 and [27, 28]) and is therefore directly relevant to the role of the oncogenic fusion protein in ARMS. This model allows us to use a physiologically relevant cell system to determine how the sole expression of the oncogenic PAX3-FOXO1 can initiate a cascade of events, both direct and downstream, over a series of proliferation events, to contribute to the initial stages of tumor development with respect to aneuploidy and overcoming aneuploidy-dependent proliferative defects. This model is in direct contrast to tumor cell lines or primary tumor samples in which it is difficult to determine whether these processes result as a “byproduct” of the final oncogenic state.

We performed mRNA and miRNA deep sequencing on total RNA isolated from three independent growths of stably transduced primary myoblasts and utilized the resulting sequencing data in large-scale comparative transcriptomic analyses, using the on-line Galaxy program or miRNAKey, respectively (see Materials and Methods). Since all stable lines were generated simultaneously and with identical methods, any artifactual effects of the process on mRNA and miRNA expression would be expected to be present in all samples and would therefore by corrected for by our differential analysis. The data used for subsequent studies were limited to 1) mRNA or miRNA displaying statistically significant differences (p < 0.05), 2) mRNA or miRNA present in both data sets being analyzed to rule out artifactual differences resulting from depth of read, and 3) mRNA or miRNA that exhibited at least 2-fold difference in expression either up- or downregulated. Finally, the biological functions of the differentially expressed mRNA or target genes of miRNA were categorized using the Onto-Express gene ontology software (http://vortex.cs.wayne.edu/projects.htm).

We found that in total PAX3-FOXO1 altered the expression of 846 genes, with 562 being upregulated and 284 being downregulated, relative to cells expressing wild-type PAX3 (data not shown). Fifty-nine of these differentially expressed genes have biological functions directly involved with multiple processes important for maintaining chromosome number and structure (Table 1) with 48/59 of these genes being downregulated. The affected genes include kinases that regulate chromosome separation and mitotic progression, cohesins, centromeric proteins, regulators of cytokinesis, components or regulators of the condensin complex, proteins involved with chromosomal segregation, and components of the mitotic spindle. Finally, six of the 59 genes that specifically contribute to promoting aneuploidy contain PAX3-FOXO1 binding sites in their proximal promoters, as previously described [29] (Table 1, carat) and six of these genes have altered gene expression levels consistent with changes seen in human tumor samples [30–33] (Table 1, pound sign).

**Table 1a:** Differentially expressed mRNA important for promoting aneuploidy by altering genes important for chromosome segregation/cytokinesis, chromosome cohesion/condensing, and mitotic progression

PROMOTION OF ANEUPLOIDY				
Gene	Function	V vs PF*	P3 vs PF	V vs P3
***Chromosome Segregation/Cytokinesis***				
ANLN	Required for cytokinesis	−2.33	−4.21	+1.89
AURKA	Regulate chromosome segregation	−2.29	−2.76	
AURKB	Regulate chromosome segregation		−5.13	
^#^CENPF	Centrosome segregation		−2.71	
CDC25B	Required for abscission in cytokinesis	+2.20		
CORO1C	Involved in cytokinesis	−2.27	−2.01	
ECT2	Nucleotide exchange factor - cytokinesis		−2.88	
ESPL1	Protease in chromosome segregation		−4.01	
^^^KLHL13	Chromosome segregation		+2.25	
MYH10	Involved in cytokinesis	−2.09	−4.58	
Pard6g	Cell division and cell polarization		−3.06	
PRC1	Regulator of cytokinesis		−2.09	
PSRC1	Required for normal chromosome segregation	−2.28	−4.15	
***Chromosome Cohesion/Condensing***				
CDC20B	Destruction of condensins		−2.71	
^^^CEP250	Centriole cohesion - chromosome segregation		−2.57	
NCAPD2	Regulatory subunit of condensin complex		−2.09	
NCAPG2	Subunit of condensin complex		−2.72	
NCAPH	Subunit of condensin complex		−3.56	
PDS5B	Regulator of cohesin		−2.18	
^#^RCC1	Regulator of chromosome condensation		−2.27	
^^^SGOL1	Involved with chromosome cohesion		−5.88	+2.76
SGOL2	Prevents premature release of cohesin		−3.41	
SMC2	Critical for mitotic chromosome condensation		−3.10	
***Mitotic Progression***				
BUB1	Mitotic checkpoint kinase		−4.71	
BUB1B	Spindle checkpoint kinase		−4.04	
CCNA2	Cyclin – regulate G2/M progression		−2.44	
CCNG1	Cyclin – regulate G2/M checkpoint	−2.19	−2.66	
CDK1	Mitotic cyclin dependent kinase	−2.46	−2.70	
CDK14	G2/M checkpoint cyclin dependent kinase		−4.56	
FoxM1	Chromosome maintenance/mitotic division		−2.45	+1.88
HELLS	Helicase in mitosis		−5.25	
^^^HMGA2	Transcriptional regulator in mitosis	−2.16	−4.35	
INCENP	Centromere protein in mitotic progression		−2.07	
NEK6	Kinase in mitotic progression		−4.05	
^#^SIRT2	Deacetylase required for mitotic exit		+2.44	

Gene name, function and fold change for each gene are listed; downregulated genes indicated by negative numbers, upregulated genes indicated by positive numbers.

* Comparisons are made between empty vector and PAX3-FOXO1 (V vs. PF), PAX3 and PAX3-FOXO1 (P3 vs. PF) or empty vector and PAX3 (V vs. P3)

^ indicates genes demonstrated to have PAX3-FOXO1 binding sites present in their proximal promoter [29]

# indicates genes that demonstrate similar trends in gene expression changes in human tumor samples [30–33].

**Table 1b:** Differentially expressed mRNA important for promoting aneuploidy by altering genes important for the segregation machinery

PROMOTION OF ANEUPLOIDY				
Gene	Function	V vs PF*	P3 vs PF	V vs P3
***Segregation Machinery***				
BRCA1	Centrosomal microtubule nucleation		−5.76	
CEP41	Centrosomal protein	+2.37	+16.62	
CEP128	Centrosomal protein		+7.62	
CEP170B	Microtubule organization	−2.19		
CKAP	Stabilizes microtubules	−2.43		
KIF11	Motor protein in centrosome separation		−4.67	
KIF20A	Motor protein in centrosome separation		−3.45	
KIF23	Kinesin – chromosome movement in division		−3.76	+2.11
^^^^#^KIF2A	Motor protein – normal mitotic progression	−2.40	−2.41	
^^^KIF3A	Kinesin – chromosome movement in division		+3.37	
KNSTRN	Mitotic spindle component		−2.39	
NDE1	Microtubule organization and mitosis	−2.22		
NIN	Important for centrosomal function		−2.94	
SDCCAG8	Centrosome associated protein	−3.41	−4.37	
SFI1	Mitotic spindle assembly		−2.23	
SPAG5	Associated with mitotic spindle		−2.61	
SUV39H1	Loss causes chromosome instability	−2.11	−3.05	
TACC3	Stabilizes mitotic spindles		−2.78	+2.16
TOP2A	Topoisomerase – chromatid separation	−2.67	−3.30	
TOPBP1	Topoisomerase binding protein	−2.01	−2.44	
TPX2	Mitotic spindle assembly factor	−2.70	−3.43	
^#^TUBB	Major component of microtubules		−2.25	
TUBB2B	Major component of microtubules		+3.26	
^#^TUBB4B	Major component of microtubules	−2.07		

Gene name, function and fold change for each gene are listed; downregulated genes indicated by negative numbers, upregulated genes indicated by positive numbers.

* Comparisons are made between empty vector and PAX3-FOXO1 (V vs. PF), PAX3 and PAX3-FOXO1 (P3 vs. PF) or empty vector and PAX3 (V vs. P3)

^ indicates genes demonstrated to have PAX3-FOXO1 binding sites present in their proximal promoter [29]

# indicates genes that demonstrate similar trends in gene expression changes in human tumor samples [30–33].

**Table 1c:** Differentially expressed mRNA important for regulating proliferative control by altering genes for proliferative transcription factors and cell cycle regulatory proteins

PROLIFERATIVE CONTROL				
Gene	Function	V vs PF*	P3 vs PF	V vs P3
***Transcription Factors***				
bcl6	Suppresses cellular proliferation		+5.94	
^#^CREB3	Promotes cellular proliferation	+2.25	+2.48	
FOXM1	Promotes entry into S- and M-phase		−2.45	+1.88
^^^^#^FOXO1	Promotes cellular proliferation	+3.16	+2.19	
FOXO4	Promotes cellular proliferation	+3.16	+3.84	
^^^^#^GADD45A	Inhibits entry into S-phase		+2.22	
HOXB9	Promotes cellular proliferation	+42.81	+9.25	
KLF5	Promotes cellular proliferation	+2.83	+2.06	
^#^MYC	Promotes cellular proliferation		+23.59	−27.09
NAB2	Negative cofactor – inhibits proliferation	+2.60		
NDN	Prevents excessive proliferation	−2.03		
^#^Nupr1	Inhibits cellular proliferation		+3.10	
PML	Promotes cellular proliferation		−4.86	
Suv39h1	Promotes cellular proliferation	−2.11	−3.05	
^#^TFDP1	Promotes E2F activity/cellular proliferation		−2.06	
UHRF1	Regulates G1/S transition		−4.00	
WRD6	G1 arrest – inhibits proliferation	−2.57		
***Cell Cycle Regulatory Proteins***				
ARID3A	Promotes E2F transcription and growth	+4.53	+4.38	
^#^BTG2	Anti-proliferative – inhibits Cyclin D1	−2.07		
CCNA2	Cyclin A2 – G1/S and G2?M progression		−2.45	
^#^CCND1	Cyclin D1 – G1 progression	−5.46	−4.32	
^#^CCND2	Cyclin D2 – G1 progression	−5.54	−4.38	
^#^CCND3	Cyclin D3 – G1 progression		+4.03	
CCNG1	Cyclin G1 – negative regulator of cell cycle	−2.19	−2.66	
CCPG1	Positive regulator of proliferation	+2.93		
CCR	Increase in G1/S – promotes proliferation		−2.57	+2.03
CDC20	Required for completion of mitosis	−2.27	−2.71	
CDC25B	Required for G2/M progression	+2.20		
CDK1	Promotes G2/M progression	−3.01	−2.71	
^^^^#^CDK6	Promotes G1 progression		−2.81	
CDK14	Promotes G1 progression		−4.56	
CDK2AP	Interacts with CDK2 – promotes proliferation	+2.48		
cdkn1a (p21)	Inhibitor of G1 progression		+2.19	
cdkn1c (p57)	Inhibitor of G1 progression	+21.71	+134.36	−5.31
cdkn2d (p19)	Inhibitor of G1 progression	+7.78	+2.46	
DBF4	Required for S-phase progression		−2.57	
^^^^#^GAS1	Block entry into S-phase		+4.26	−3.63
Rb1	Tumor suppressor – inhibits proliferation		+2.10	−2.53
Mdm2	Facilitates G1/S phase transition	−2.11		
NEK6	Required for M-phase progression		−4.06	

Gene name, function and fold change for each gene are listed; downregulated genes indicated by negative numbers, upregulated genes indicated by positive numbers.

* Comparisons are made between empty vector and PAX3-FOXO1 (V vs. PF), PAX3 and PAX3-FOXO1 (P3 vs. PF) or empty vector and PAX3 (V vs. P3)

^ indicates genes demonstrated to have PAX3-FOXO1 binding sites present in their proximal promoter [29]

# indicates genes that demonstrate similar trends in gene expression changes in human tumor samples [30–33].

**Table 1d:** Differentially expressed mRNA important for regulating proliferative control by altering genes for growth factors and proliferative receptors

PROLIFERATIVE CONTROL				
Gene	Function	V vs PF*	P3 vs PF	V vs P3
***Growth Factors/Development***				
CGREF1	Ca+2 binding inhibitor of cellular proliferation		−3.10	+2.44
CSF1	Promotes cellular proliferation	−5.94	−3.29	
^#^CYR61	Promotes cellular proliferation	−3.29		
hdgfrp3	May promote cellular proliferation		+6.15	
^#^IFITM1	Suppresses proliferation	−2.68		−2.89
^#^Igf2	Growth promoting hormone		+19.97	−3.72
^#^IGFBP2	Inhibits IGF-dependent proliferation	−8.63		
Igfbp3	Interacts with and stabilizes IGF		+4.35	−1.74
^#^Igfbp5	Interacts with and stabilizes IGF	+8.28	+50.91	−5.70
RACGAP1	Promotes cellular proliferation		−2.68	
^^^SHB	Promotes IGF-dependent cellular proliferation	+2.93	+3.23	
^#^SMAD3	Promotes cellular proliferation	−5.50		
SMO	Promotes cellular proliferation		−2.38	
^#^TgfB3	Anti-proliferative effect		+2.77	−2.75
***Receptor/Signal Transduction***				
^^^^#^ABI1	Negative regulator of proliferation	−2.41		
ADRA1B	Adranergic receptor – inhibits proliferation		−4.89	+3.34
^#^AKAP12	Suppresses proliferation	−7.94		−3.86
AXL	Promotes cellular proliferation	−6.06	−4.29	
EGFR	Epidermal growth factor receptor		−3.32	+4.82
EPS8	Involved in promoting EGF pathway		−9.32	+3.58
Erbb3	HER3 –growth factor receptor	+3.10	+6.23	−4.06
^^^^#^FGFR4	Receptor for FGF19 – promotes proliferation	+6.11		
GAREM	Promotes EGF-receptor proliferation	+15.78	+4.96	
GHR	Promotes cellular growth		−4.66	+2.28
^#^Grb10	Negative regulator of proliferation		+7.11	−11.47
^^^IGF1R	IGF1 growth factor receptor		+2.69	−2.13
IL6ST	Promotes cellular proliferation	+2.77	+3.05	
^^^IRS1	Involved in insulin/IGF signaling	−2.14		
^^^^#^MET	Hepatocyte growth factor receptor		+2.91	−3.73
NOTCH2	Promotes myoblast proliferation		+2.38	
SPHK2	Sphingosine kinase – promotes proliferation	+2.16		
Tgfbr1	Anti-proliferative effects		+2.25	

Gene name, function and fold change for each gene are listed; downregulated genes indicated by negative numbers, upregulated genes indicated by positive numbers.

* Comparisons are made between empty vector and PAX3-FOXO1 (V vs. PF), PAX3 and PAX3-FOXO1 (P3 vs. PF) or empty vector and PAX3 (V vs. P3)

^ indicates genes demonstrated to have PAX3-FOXO1 binding sites present in their proximal promoter [29]

# indicates genes that demonstrate similar trends in gene expression changes in human tumor samples [30–33].

**Table 1e:** Differentially expressed mRNA important for regulating proliferative control by altering genes for proliferative enzymes and miscellaneous proliferative proteins

PROLIFERATIVE CONTROL				
Gene	Function	V vs PF*	P3 vs PF	V vs P3
***Enzymatic Activity***				
^#^ADAMTS1	Metalloproteinase – promotes proliferation	+22.01	+3.63	+5.66
BRCA1	Tumor suppressor – inhibits proliferation		−5.78	
^#^DUSP1	Phosphatase – inhibits proliferation	−3.61		−2.31
DUSP4	Phosphatase – inhibits proliferation	−3.07	−2.43	
DUSP10	Phosphatase – inhibits proliferation			−2.20
^^^^#^DYRK	Kinase – inhibits cellular proliferation		+2.75	−3.34
PDIA4	Protein disulfide isomerase – IGFR recycler			+2.73
PPP6C	Phosphatase – restricts G1/S progression		+2.57	
^^^PRUNE	Phosphodiesterase – promotes proliferation		+3.32	−2.66
PTPRK	Negative regulator of EGFR		−7.89	
TENC1	Negative regulator of Akt		+2.45	
^#^TIMP2	Inhibits cellular proliferation	−2.68		
***Other***				
^#^CAV1	Antiproliferative – downregulates Cyclin Da		+5.31	−2.95
CRLF3	Negative regulator of cell cycle progression		−3.10	
EPB41L3	Suppresses proliferation	−6.32	−3.20	
FOSL1	Promotes cellular proliferation	−5.21	−4.69	
Gpnmb	Inhibits cellular proliferation		−14.52	+3.32
^#^NPM1	Promotes cellular proliferation		−2.31	
PHF10	Chromatin remodeler – promotes proliferation	+2.13	+2.99	
SDC1	Promotes proliferation	−2.43	−2.33	
UBN1	Chromatin remodeler – promotes senescence		+2.10	

Gene name, function and fold change for each gene are listed; downregulated genes indicated by negative numbers, upregulated genes indicated by positive numbers.

* Comparisons are made between empty vector and PAX3-FOXO1 (V vs. PF), PAX3 and PAX3-FOXO1 (P3 vs. PF) or empty vector and PAX3 (V vs. P3),

^ indicates genes demonstrated to have PAX3-FOXO1 binding sites present in their proximal promoter [29]

# indicates genes that demonstrate similar trends in gene expression changes in human tumor samples [30–33].

In our miRNA deep sequencing analysis we found a total of 104 miRNAs whose expression changed in a PAX3-FOXO1-dependent manner, 61 of which are downregulated and 43 of which are upregulated (data not shown). Using miRTarBase [34] we found that out of these 104 total miRNA, 19 have validated target genes important for maintaining chromosome number and integrity (10 downregulated, 9 upregulated, Table 2), with validation on miRTarBase by at least two independent experimental methods. In addition to these 19 validated targets, we found using the TargetScan database that 22 additional miRNAs (17 downregulated, 5 upregulated, Table 2) are highly predicted to target genes important for chromosome number and integrity, based on their predicted efficacy of targeting (context score ≥ 85%) [35, 36] or probability of conserved targeting (P_CT_) [37] [P_CT_ ≥ 0.8], as previously described [38], with several of the genes identified by TargetScan being validated targets in MirTarBase. Combined, the differentially expressed miRNAs target 56 additional genes that promote aneuploidy with many of these genes being targeted by multiple differentially expressed miRNA.

**Table 2a:** Differentially expressed miRNA that target genes important for promoting aneuploidy — miRNA downregulated by PAX3-FOXO1 relative to empty vector

PROMOTION OF ANEUPLOIDY					
iR	Target	Gene Function	V vs. PF	P3 vs. PF	V vs. P3
*10a-5p	HDAC4	Regulates chromosome segregation	−333.33	−200.00	
*1a-3p	Calm1 Calm2 HDAC4 Cdc42	Regulates progression of cytokinesis Regulates progression of cytokinesis Regulates chromosome segregation Spindle microtubule attachment	−18.50	−11.11	
*376a-3p	TTK	Chromosome alignment at centromere	−11.08	−12.67	
*433-3p	*CEP135*	*Centrosomal protein*	−5.52	−3.97	
543-3p	*SIRT1*	*Involved in chromosome maintenance*	−4.50	−3.31	
*133a-3p	Cdc42 HDAC4	Spindle microtubule attachment Regulates chromosome segregation	−3.72	−3.62	
148b-3p	*CDC25B*	*Induces mitotic progression*	−3.44	−3.70	
*19a-3p	*CEP350* *MAPRE2*	*Anchors microtubules to centrosome* *Anchors microtubules to centrosome*	−2.87	−2.84	
*351-5p	*NINL* *SUV39H1*	*Mitotic spindle assembly* *Methylase important for segregation*	−2.43	−2.12	
3099-3p	*KIF3B*	*Tethers chromosomes to spindle pole*	−2.36	−4.20	
133b-5p	*Myh9*	*Important for cytokinesis*	−2.26	−2.89	
504-5p	*CEP170*	*Centrosomal protein*	−2.09	−3.87	
*335-5p	*CHFR* *d4*	*Regulates entry into mitosis* *Regulates chromosome stability*	−12.50	−37.04	+2.95
486-3p	PTEN	Chromosome stability	−6.70	−13.20	+1.97
*128-3p	*NEK6* *PARD6B* *PDS5B*	*Required for chromosome segregation* *Involved in asymmetrical cell division* *Important for sister chromatid cohesion*	−2.17	−4.85	+2.32
*339-5p	*MAPRE1*	*Anchors microtubules at centrosome*	−1.93	−4.24	+2.19
*148a-3p	CamK2 *CDC25B* *CCNF* *PTEN* *RCC2*	Spindle depolarization *Induces mitotic progression* *Inhibitor of centrosome reduplication* *Chromosome stability* *Regulates chromosome condensation*	−4.41		−2.98
*29a-3p	HDAC4 MCL1 *Tubb2B* *KIF3B* *CEP68* *PTEN* *CDC42BPA* *MAPRE2*	Regulates chromosome segregation Inhibits BCL2 and apoptosis *Component of microtubules* *Tethers chromosomes to spindle pole* *Centrosomal protein* *Chromosome stability* *Regulates CDC42* *Anchors microtubules to centrosome*	−2.65		
3968	*ESCO2*	*Establishment of sister chromatid cohesion*	−2.53		
*322-5p	Arl2 *CDC27*	Regulates centrosome integrity *Regulates mitotic progression*	−2.42		
*9-5p	SIRT1 *CCNG1* *CDC14B* *CEP350*	Involved in chromosome maintenance *Associated with G2/M arrest* *Controls exit of mitosis* *Centrosomal protein*		−5.75	+9.73
133b-3p	Pitx3	Important for mitotic activity		−4.83	+5.74
*486-5p	PTEN	Chromosome stability		−2.80	+2.09
*206-5p	*HDAC4*	*Regulates chromosome stability*		−2.63	+2.31

The microRNA, target gene name, function, and fold change for each miRNA are listed; downregulated microRNA indicated by negative numbers, upregulated miRNA indicated by positive numbers. Genes and functions listed in normal font indicate validated targets; genes and functions listed in italics indicate genes highly predicted to be targets of the indicated microRNA. Comparisons are made between empty vector and PAX3-FOXO1 (V vs. PF), PAX3 and PAX3-FOXO1 (P3 vs. PF) or empty vector and PAX3 (V vs. P3)

* indicates miRNA that target genes involved with the promotion of aneuploidy and in proliferative control.

**Table 2b:** Differentially expressed miRNA that target genes important for promoting aneuploidy — miRNA upregulated by PAX3-FOXO1 relative to empty vector

PROMOTION OF ANEUPLOIDY					
miR	Target	Gene Function	V vs. PF	P3 vs. PF	V vs. P3
615-3p	MAPT	Determines polarity of the centrosome	+30.45	+6.08	+5.01
*31-3p	RHOA	Signaling protein important for cytokinesis	+4.19	+2.04	+2.05
*196a-5p	Hmga2 RCC2	Chromosome condensation – G2/M phase Regulator of chromosome condensation	+24.39	+19.33	
92a-1-5p	TACC2	Organizes centrosomal microtubules	+3.35	+3.55	
*301a-3p	MDM4 CENPO	Regulates chromosome stability Necessary for chromosome segregation	+3.07	+2.33	
*16-5p	Arl2 MDM4 G2E3	Regulates centrosome integrity Regulates chromosome stability Important for mitotic progression	+2.07	+2.56	
*222-3p	PTEN	Chromosome stability	+2.78	+7.02	−2.52
*221-3p	PTEN	Chromosome stability	+2.10	+7.02	−2.52
*30c-2-5p	RCC2 CEP350	Regulator of chromosome condensation Anchors microtubules at centrosome		+3.55	−2.32
20a-5p	Mapk4 PTEN	Important for cytokinesis Chromosome stability		+2.01	
*130b-3p	MAP4	Important for chromosome segregation	+3.39		+2.20
421-3p	ARHGEF9	Regulates spindle microtubule attachment	+3.22		+3.27
*206-3p	Hdac4 Tppp CORO1C	Regulates chromosome segregation Mitotic spindle assembly Potential role in cytokinesis	+2.42		+4.49
*15b-5p	Arl2 CDC25A	Regulates centrosome integrity Induces mitotic progression	+2.86		
183-5p	KIF2A	Microtubule associated protein – mitosis	+2.05		
*Let-7g-5p	HMGA2	Transcriptional regulator in mitosis		+2.04	−2.15

The microRNA, target gene name, function, and fold change for each miRNA are listed; downregulated microRNA indicated by negative numbers, upregulated miRNA indicated by positive numbers. Genes and functions listed in normal font indicate validated targets; genes and functions listed in italics indicate genes highly predicted to be targets of the indicated microRNA. Comparisons are made between empty vector and PAX3-FOXO1 (V vs. PF), PAX3 and PAX3-FOXO1 (P3 vs. PF) or empty vector and PAX3 (V vs. P3)

* indicates miRNA that target genes involved with the promotion of aneuploidy and in proliferative control.

**Table 2c:** Differentially expressed miRNA that target genes important for proliferative control — miRNA downregulated by PAX3-FOXO1 relative to empty vector

PROLIFERATIVE CONTROL					
miR	Target	Gene Function	V vs. PF	P3 vs PF	V vs P3
*10a-5p	HDAC4	Histone deacetylase 4 – pro-proliferative	−333.33	−200.00	
143-3p	Kras Ptn	G-protein coupled receptor Secreted growth factor – mitogenic	−22.22	−17.85	
*1a-3p	HDAC4 IGF1 Igf1R PDGFA TIMP3	Pro-proliferative Insulin like growth factor - proliferative Insulin like growth factor receptor Growth factor – promotes proliferation Inhibits cellular proliferation	−18.50	−11.11	
133a-5p	INSR FGF1 FGFR1	Insulin receptor – proliferative Growth factor ligand – proliferative Growth factor ligand – proliferative	−16.76	−12.67	
*376a-3p	CDK2 IGF1R	Cyclin dependent kinase – G1/S Insulin like growth factor 1 receptor	−11.08	−13.56	
*433-3p	GRB2	EGF-dependent proliferation	−5.52	−3.97	
*133a-3p	Spry1 CCND2 Igf1R EGFR	Antagonist of the FGF pathway Cyclin D2 – cell cycle regulation Insulin like growth factor 1 receptor Epidermal growth factor receptor	−3.72	−3.62	
362-3p	CDKN1A Rb1 HBEGF	Cyclin dependent kinase inhibitor (p57) Cell cycle regulatory protein Growth factor with EGFR and ERRB2	−3.67	−3.21	
*19a-3p	CDKN1A CCND1 MDM4 MAPK1 HDAC4 GRB10 IGFBP3 CCND2	Cyclin dependent kinase inhibitor (p57) Cyclin D1 – G1/S progression Promotes cellular proliferation Kinase – promotes proliferation Histone deacetylase 4 – proliferative Negative regulator of proliferation Interacts with and stabilizes IGF Cyclin D2 – promotes cell cycle	−2.87	−2.84	
*34b-5p	E2F3	Promotes cell cycle progression	−2.60	−2.43	
*351-5p	E2F2	Promotes cell cycle progression	−2.43	−2.12	
*335-5p	Rb1	Tumor suppressor – cell cycle regulator	−12.50	−37.04	+2.95
145a-5p	Hoxa9 IRS1 Kras CCND2	Homeobox transcription factor Insulin signaling pathway Protooncogene – proliferative Cylcin D2 – cell cycle regulation	−7.59	−14.83	+1.95
335-3p	IGF1R	IGF1 receptor – pro-proliferative	−5.85	−15.87	+2.72
*128-3p	Trim71 FoxP1 c-Met IRS1 SOS1	E3-ubiquitin ligase –G1/S transition Inhibits proliferation Hepatocyte growth factor receptor Involved with insulin signaling Promotes cellular proliferation	−2.17	−4.85	+2.32
*339-5p	Kdm6b	Histone demethylase – pro-proliferative	−1.93	−4.24	+2.19
*148a-3p	Kdm6b Dnmt1 ERBB3 CDC25B CDKN1B E2F7	Histone demethylase – pro-proliferative DNA methyltransferase – pro-proliferative Human epidermal growth factor receptor Required for G2/M progression Inhibitor of G1 progression Promotes cell cycle progression	−4.41		−2.98
*181c-5p	KRAS E2F7	Promotes cell cycle progression Proto-oncogene – proliferative	−2.34		−3.95
149-3p	E2F1 MYBL2	Cell cycle regulatory transcription factor Proliferative transcription factor	−2.34	+2.99	−7.04
*29a-3p	CDK6 PDGFRB PDGFA FOXO3 PDGFB	Promotes G1 progression Promotes proliferation Promotes proliferation Promotes proliferation Promotes proliferation	−2.65		
*322-5p	CDC27	Promotes M-phase progression	−2.42		
340-5p	MET	Hepatocyte growth factor receptor	−2.06		
450a-5p	DUSP10	Negative regulator of proliferation		−2.03	
*9-5p	CDKN1A Hes1 FGF5 BRAF CDC25A IGFBP3 FOXO1 FOXO3 HDAC5	Cyclin dependent kinase inhibitor (p21) Promotes proliferation Growth factor – promotes proliferation Pro-proliferative kinase Cell Cycle regulatory protein Interacts with and stabilizes IGF Promotes cellular proliferation Promotes cellular proliferation Promotes cellular proliferation		−5.75	+9.73
*486-5p	FOXO1	Promotes cellular proliferation		−2.80	+2.09
*206-5p	HDAC4	Histone deacetylase 4 – pro-proliferative		−2.63	+2.31
345-5p	CDKN1A IGFBP5	Cyclin dependent kinase inhibitor (p21) Interacts with and stabilizes IGF		−2.39	+3.09
23b-3p	Hes1 MET	Promotes proliferation Hepatocyte growth factor receptor		−2.10	+2.19

The microRNA, target gene name, function, and fold change for each miRNA are listed; downregulated microRNA indicated by negative numbers, upregulated miRNA indicated by positive numbers. Genes and functions listed in normal font indicate validated targets; genes and functions listed in italics indicate genes highly predicted to be targets of the indicated microRNA. Comparisons are made between empty vector and PAX3-FOXO1 (V vs. PF), PAX3 and PAX3-FOXO1 (P3 vs. PF) or empty vector and PAX3 (V vs. P3)

* indicates miRNA that target genes involved with the promotion of aneuploidy and in proliferative control.

**Table 2d:** Differentially expressed miRNA that target genes important for proliferative control — miRNA upregulated by PAX3-FOXO1 relative to empty vector

PROLIFERATIVE CONTROL					
miR	Target	Gene Function	V vs. PF	P3 vs PF	V vs P3
*31-3p	ERBB2 E2F1	HER2 growth factor receptor Cell cycle regulatory transcription factor	+4.19	+2.04	+2.05
*196a-5p	CDKN1B HMGA2 ING5	Cyclin dependent kinase inhibitor Promotes myoblast proliferation Inhibitor of growth – p53 pathway	+24.39	+19.33	
*301a-3p	E2F2 E2F7 ERBB4 JARID2 MAPK1 MDM4 PTEN	Promotes cell cycle progression Promotes cell cycle progression HER4 – growth factor receptor Inhibits proliferation Promotes proliferation Promotes proliferation Inhibits proliferation	+3.07	+2.33	
*16-5p	Wnt3a CCND1 Mdm4 Jun CCNE1 G2E3 FGF7 FGF2	Promotes proliferation Cylcin D1 – G1 progression Promotes proliferation Transcription factor Cyclin E1 – G1/S transition G2/M-specific E3 Ubiquitin ligase Growth factor – pro-proliferative Growth factor – pro-proliferative	+2.07	+2.56	
*222-3p	CDKN1B	Cyclin-dependent kinase inhibitor	+2.78	+7.02	−2.52
*155-5p	Jarid2 Myb FGF7 Fos	Inhibits proliferation Regulates proliferation Fibroblast growth factor ligand Transcription factor	+2.18	+5.54	−2.53
*221-3p	CDKN1B	Cyclin dependent kinase inhibitor (p57	+2.10	+6.97	−3.31
92b-5p	CDKN1C	Cyclin dependent kinase inhibitor (p16)		+3.75	−2.77
*30c-2-5p	HDAC4 MAPKBP1 ATF1 KRAS IRS2 IGF1R FOXO3	Promotes proliferation Promotes proliferation Promotes proliferation Proto-oncogene – proliferative Insulin signaling pathway Insulin receptor – proliferative Promotes proliferation		+3.55	−2.32
181c-3p	E2F7	Promotes cell cycle		+2.74	−3.77
*30a-5p	Egfr RUNX2 SOS IGF1R	Growth factor receptor – proliferative Inhibits cellular proliferation Promotes cellular proliferation Growth factor receptor		+2.44	−2.50
99a-5p	FGFR3 IGF1R FOXO4	Growth factor receptor Growth factor receptor Proliferative transcription factor		+2.05	−2.45
*Let-7g-5p	Myc IGFBP1 CDKN2A	Proliferative transcription factor Inhibits IGF-dependent proliferation Cyclin dependent kinase inhibitor (p16)		+2.04	−2.15
*130b-3p	MET MAPK1 JARID2 E2F2 E2F7 ERBB4 PTEN MDM4	Hepatocyte growth factor receptor Kinase – promotes proliferation Inhibits proliferation Promotes cell cycle progression Promotes cell cycle progression HER4 – growth factor receptor Inhibits proliferation Promotes proliferation	+3.39		+2.20
*206-3p	HDAC4 Spry1 Id1 TIMP3	Promotes proliferation Antagonist of the FGF pathway Promotes proliferation Inhibits proliferation	+2.42		+4.49
*15b-5p	CCNE2 CCND1 MYB	Cyclin E – G1/S phase progression Cyclin D – G1/S phase progression Promotes proliferation	+2.86		
*30b-5p	CCNE2	Cyclin E – G1/S phase progression	+1.98		

The microRNA, target gene name, function, and fold change for each miRNA are listed; downregulated microRNA indicated by negative numbers, upregulated miRNA indicated by positive numbers. Genes and functions listed in normal font indicate validated targets; genes and functions listed in italics indicate genes highly predicted to be targets of the indicated microRNA. Comparisons are made between empty vector and PAX3-FOXO1 (V vs. PF), PAX3 and PAX3-FOXO1 (P3 vs. PF) or empty vector and PAX3 (V vs. P3)

* indicates miRNA that target genes involved with the promotion of aneuploidy and in proliferative control.

To determine if the PAX3-FOXO1-dependent mRNA and miRNA changes translate into differences in chromosome number and structure, we performed a cytogenetic analysis of proliferating primary myoblasts (Table 3A and Figure 1C) and found that the majority (48/62 cells - 77.4%) of cells transduced with empty vector contain the normal complement of 40 (2N) or 80 (4N) chromosomes, which is consistent with previous reports for the presence of diploidy and tetraploidy in proliferating myoblasts [39]. In a similar manner, a majority of cells (46/50 cells - 92%) ectopically expressing PAX3 contained the normal complement of chromosomes, demonstrating that the process of transduction and ectopic expression of protein does not affect chromosome number or structure. In contrast, nearly all of the cells stably expressing PAX3-FOXO1 (93/103 cells - 90.3%) had hypodiploid (<2N) or hyperdiploid/hypotetraploid (>2N to <4N) chromosome numbers. The numbers of chromosomes seen in individual cells, along with the variation of chromosome numbers between cells correlates will with results seen in ARMS primary tumor samples [11, 15–18].

**Table 3A:** Quantification of PAX3-FOXO1-dependent aneuploidy on cells stably transduced with empty vector (Vector), PAX3, PAX3-FOXO1, or the PAX3-FOXO1 phosphomutants (S201A, S205A, and S209A)

Sample		Chromosome Number				
		Hypodiploid	Diploid	Hyperdiploid/Hypotetraploid	Tetraploid	Hypertetraploid
	Number cells analyzed	<2N (<40)	2N (40)	>2N to <4N (41 – 79)	4N (80)	>4N (>80)
Vector	62	7 (11.3%)	25 (40.3%)	7 (11.3%)	23 (37.1%)	0 (0.0%)
PAX3	50	2 (4.0%)	34 (68.0%)	2 (4.0%)	12 (24.0%)	0 (0.0%)
PAX3-FOXO1	103	51 (49.5%)	6 (5.8%)	42 (40.8%)	4 (3.9 %)	0 (0.0%)
S201A	64	29 (45.3%)	5 (7.8%)	27 (42.2%)	2 (3.1%)	1 (1.5%)
S205A	69	13 (18.8%)	3 (4.3%)	45 (65.2%)	4 (5.8%)	4 (5.8%)
S209A	57	18 (31.6%)	2 (3.5%)	29 (50.8%)	0 (0.0%)	8 (14.0%)

The chromosome number for individual cells was determined and classified as hypodiploid (<2N), diploid (2N), hyperdiploid/hypotetrapoid (>2N to <4N), tetraploid (4N) or hypertetraploid (>4N)

**Table 3B:** Quantification of PAX3-FOXO1-dependent chromosomal structural abnormalities

Sample	Number cells analyzed	Sister Chromatid Dissociation		Telomere Association		Double Minutes	
		Number of Cells	Range of Events/Cell	Number of Cells	Range of Events/Cell	Number of Cells	Range of Events/Cell
Vector	62	1 (1.6%)	2	2 (3.2%)	2	0 (0.0%)	0
PAX3	50	2 (4.0%)	2	2 (4.0%)	2	0 (0.0%)	0
PAX3-FOXO1	103	16 (15.5%)	1 – 5	17 (16.5%)	1 – 16	4 (3.9%)	5 – 20
S201A	64	6 (9.4%)	2 – 6	21 (32.8%)	2 – 18	4 (6.3%)	6 – 12
S205A	69	9 (13.0%)	1 – 3	20 (28.9%)	1 – 4	5 (7.2%)	1 – 62
S209A	57	7 (12.3%)	5 – 63	6 (10.5%)	1 – 3	4 (7.0%)	5 – 40

Metaphase chromosome analysis on cells stably transduced with empty vector (Vector), PAX3, PAX3-FOXO1, or the PAX3-FOXO1 phosphomutants (S201A, S205A, or S209A). The number of cells containing the indicated structural abnormality along with a range of the number of occurrences of the abnormality within each cell is listed.

Further, we observed an increased number of disrupted chromosomal structures in cells stably expressing PAX3-FOXO1 relative to the negative control cells (vector and PAX3). We noted 16 cells (15.5%) with sister chromatid dissociation (Figure 1C, right panel, solid arrows), 17 cells (16.5%) with telomere association, and 4 cells (3.9%) with double minutes (Table 1B) with only two of these cells having the presence of both sister chromatid dissociation and telomere association. Although some chromosomal disruptions were observed in the control cells, these events were minimal (incidence <4%) and did not include the presence of double minutes (Table 3B).

We previously published that PAX3-FOXO1 is phosphorylated at three independent sites [27, 28, 40] and phosphorylation contributes to ARMS oncogenic phenotypes in an *in vitro* cellular model [26]. To determine how phosphorylation at these sites affects PAX3-FOXO1-dependent changes in chromosome number and structure, we utilized mutants in which each individual site was mutated to a phospho-incompetent alanine (S201A, S205A, or S209A) [26]. A Western blot analysis of mouse primary myoblasts stably transduced with these mutants demonstrates that all mutants were expressed to levels equivalent to that of wild-type PAX3-FOXO1 (Figure 1B).

Cytogenetic analysis demonstrated that similar to wild-type PAX3-FOXO1, a majority of cells stably expressing S201A [57/64 cells - 89.1%], S205A [62/69 cells - 89.8%], and S209A [55/57 cells - 96.5%] had chromosome numbers <2N or >2N to <4N with an increase in the number of hypertetraploid cells (>4N) for all three samples (Table 3A). We also observed similar chromosomal abnormalities for each of the phopsho-incompetent mutants relative to wild-type PAX3-FOXO1 (Figure 1D); however, the individual phosphorylation events seem to impact these chromosomal aberrations differently (Table 3B). While all three mutants have a similar percentage of cells containing sister chromatid dissociation relative to wild-type PAX3-FOXO1, the expression of S209A increased the number of individual events within each cell, with some cells having over 60 dissociations. Although both S201A and S205A increased the percentage of cells with telomere association, loss of phosphorylation at S205 or S209 decreased the number of individual events within each cell relative to PAX3-FOXO1. Finally, double minutes were present in a similar percentage of cells expressing the fusion protein as either wild type or mutant. However, cells stably expressing the S205A mutant had an increased number of double minutes per cell relative to the wild-type fusion protein. Taken together, these results are the first to demonstrate that the presence of an oncogenic fusion protein, PAX3-FOXO1, is sufficient to promote aneuploidy and disrupt chromosome structure and that phosphorylation contributes to this state.

### PAX3-FOXO1 overrides cell cycle inhibition to enhance cellular proliferation

One of the initial cellular responses to aneuploidy is an attenuation of proliferation [41]. An examination of our comparative transcriptomic analysis revealed that of the 846 differentially expressed mRNA, 93 (nearly 11%) have biological functions important for cellular proliferation (Table 1), twelve of which have previously described PAX3-FOXO1 binding sites in their proximal promoter (Table 1, carat) [29], and 30 had altered gene expression levels consistent with changes seen in human tumor samples [30–33] (Table 1, pound sign). We also found 13 of the downregulated and 9 of the upregulated miRNAs are experimentally validated on miRTarBase to target mRNA whose biological function is important for proliferative control. Further, 15 of the downregulated and 9 of the upregulated miRNAs are highly predicted to target growth regulatory genes (Table 2), based on their predicted efficacy of targeting or probability of conserved targeting, as described above.

**Figure 2:**
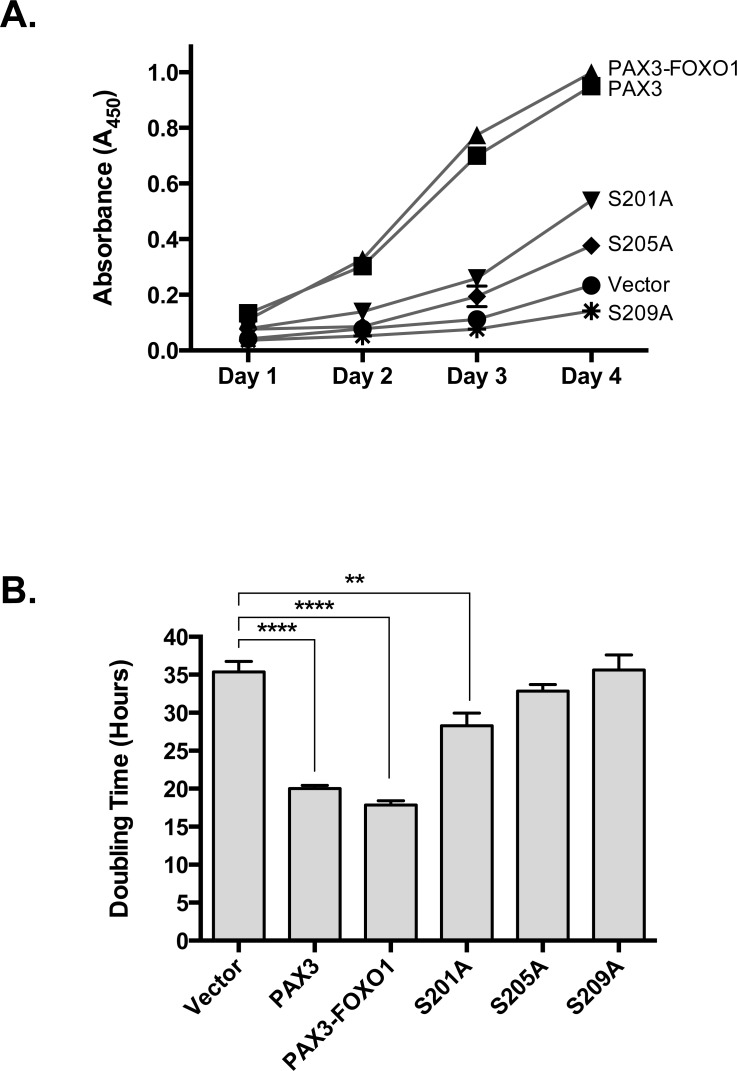
Stably transduced primary myoblasts (vector, PAX3, wild-type PAX3-FOXO1, or PAX3-FOXO1 phosphomutant) were plated, grown for four days, growth determined using the CCK-8 cell counting kit and plotted as a function of time (A) from which doubling times were determined, as described in the Materials and Methods Error bars represent the standard deviation from three independent determinations and *P*-values were computed using non-parametric two-way ANOVA analyses comparing each treatment condition to the empty vector negative control. (**P = 0.005, ****P < 0.0001).

Consistent with the predicted cellular response to the aneuploid state, we found that of the 93 alternatively expressed mRNA, 23 are cell cycle regulatory genes and include decreases in cyclins and their related cyclin dependent kinases and increases in the expression of cyclin dependent kinase inhibitors (Table 1). Further, we found 11 of the downregulated and 12 of the upregulated miRNAs also affect cell cycle regulatory proteins in a manner consistent with the attenuation of growth. To determine how these changes translate into effects on cellular growth, we determined doubling times of primary myoblasts stably transduced with empty vector, PAX3, or PAX3-FOXO1. We found the doubling time of primary myoblasts transduced with empty vector to be approximately 35 hours and that the stable expression of PAX3 enhanced the growth rate by reducing the doubling time to approximately 20 hours (Figure 2). Primary myoblasts stably expressing PAX3-FOXO1 also had a reduced doubling time of approximately 20 hours (Figure 2), a result that seems to be in contrast to the presence of aneuploidy in these cells and the observed changes in cell cycle regulatory mRNA and miRNA. Further, we determined proliferation rates on primary myoblasts stably expressing the PAX3-FOXO1 phospho-mutants. We found that although cells expressing S201A have an enhanced proliferation rate relative to the negative control, the rate is significantly slower than for cells stably expressing wild-type PAX3-FOXO1 (Figure 2). The stable expression of S205A and S209A resulted in proliferation rates that were indistinguishable from the empty vector negative control.

To better understand this result, we examined our mRNA and miRNA comparative transcriptomic analyses for alterations in mRNA and miRNAs whose biological functions may affect growth independent of cell cycle regulatory proteins. We found that the expression of PAX3-FOXO1 is sufficient to alter the mRNA for multiple growth factors (14 genes) and growth factor receptors (18 genes) in a manner that is in direct alignment with our observed increased proliferation rate. These include the growth factor receptors c-MET [22], and IGF1R [20] (both direct targets of PAX3-FOXO1), FGFR4, Erbb3 (HER3), IL6ST, and the receptor NOTCH2, which in certain cell types enhances proliferation. We also found the increased expression of many of the associated growth factors including IGF2, IGFBP3, and IGFBP5 and several proliferative transcription factors (17 genes), including CREB3, FOXO4, HOXB9, and Myc (Table 1). Further, most of the miRNA have validated or predicted targets that are growth factor receptors, growth factors, or proliferative transcription factors (Table 2). Interestingly, 18 of the downregulated and 13 of the upregulated miRNAs target genes important for both aneuploidy and the regulation of proliferative control. Taken together, these results demonstrate that the expression of an oncogene is sufficient to override aneuploid-dependent attenuation of growth by globally altering the expression of growth factor related mRNA and miRNA.

### Phosphorylation contributes to PAX3-FOXO1-dependent differential gene expression

**Figure 3:**
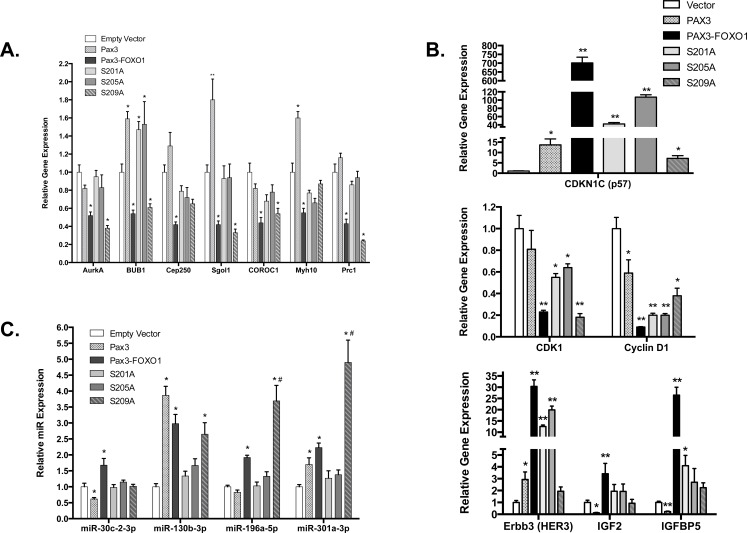
Quantitative RT-PCR analysis for aneuploidy mRNA (A), proliferation mRNA (B) and miRNA (C) Total RNA was isolated from stably transduced cells [empty vector (white), PAX3 (stippled), PAX3-FOXO1 (black), or the phosphomutants S201A (light gray), S205A (medium gray), or S209A (hashed gray). Quantitative RT-PCR was performed using primers specific for the indicated mRNA **A.** and **B.** or microRNA **C.**, as described in the Materials and Methods. Error bars represent the standard deviation from three independent determinations and P-values were computed using non-parametric two-way ANOVA analyses. The asterisk indicates statistical comparisons between empty vector and each sample (*P = 0.001, **P < 0.0001). The hash-tap indicates comparisons between wild-type PAX3-FOXO1 and the sample (#*P* = 0.001).

To determine how phosphorylation of PAX3-FOXO1 affects the expression of genes important for maintaining chromosome number and structure, we performed a qRT-PCR analysis on a subset of genes with fusion protein-dependent altered expression. We tested genes in chromosome segregation (AurkA), chromosome cohesion (BUB1, Cep250, and Sgol1), and cytokinesis (COROC1, Myh10, and Prc1). We observed changes in gene expression consistent with our mRNA deep sequencing results, in which the presence of PAX3-FOXO1 promotes a significant decrease in the expression of all seven genes (Figure 3A). Further, with the exception of Myh10 and Cep250, we found inhibiting the phosphorylation of PAX3-FOXO1 at Ser201 or Ser205 corrected these decreases, with gene expression levels equivalent to the empty vector or PAX3 controls (Figure 3A). However, loss of phosphorylation at Ser209 was unable to correct these decreases and in fact promoted a further 2-fold decrease in the expression of Prc1.

A qRT-PCR analysis on a subset of proliferation genes, including cell cycle regulatory genes (CDKN1C, CDK1, and Cyclin D1) and growth factor genes (Erbb3, IGF2, and IGFBP5) once again showed PAX3-FOXO1-dependent changes consistent with our mRNA deep sequencing results (Figure 3B). We found the stable expression of the phospho-incompetent mutants also increased (CDKN1C) or decreased (CDK1 and Cyclin D1) cell cycle regulatory gene expression relative to the negative control. However, with the exception of CDK1 in cells expressing the S209A mutant, these changes were much less severe relative to wild-type PAX3-FOXO1. With respect to growth factor related genes, we found that the S209A mutant reduced the expression of all three genes to levels indistinguishable from the negative control. Further, the expression of S201A and S205A also altered the expression of IGF2 and IGFBP5 that trended toward background levels, but had little effect on the expression of Erbb3.

We performed a similar quantitative analysis on a select set of miRNAs, chosen for their ability to target genes important for the promotion of aneuploidy and for the regulation of proliferation. We found changes in miRNA expression consistent with our deep sequencing results (Figure 3C). As with our mRNA results, we found that inhibiting phosphorylation of PAX3-FOXO1 at Ser201 or Ser205 returned miRNA expression levels to that of the empty vector or PAX3 controls (Figure 3C). While loss of phosphorylation at Ser209 was able to correct the altered miRNA expression for miR30c-2-5p, S209A had no effect on the altered expression of miR130b-3p. Interestingly, the expression of S209A resulted in a 2- or 4-fold increase in the expression of miR-196a-5p and miR301a-3p, respectively, relative to wild-type PAX3-FOXO1 (Figure 3C). Taken together, our results demonstrate that phosphorylation contributes to the altered expression of genes and miRNAs important for the PAX3-FOXO1-dependent promotion of aneuploidy, aberrant chromosome structure, and regulation of growth.

## DISCUSSION

Aneuploidy is common in solid tumors, which given the enhanced proliferative activity of transformed or malignant cells, suggests that these cells have acquired mechanisms to overcome proliferative defects associated with this state. In this report we demonstrate for the first time that the presence of an oncogenic protein, PAX3-FOXO1, is sufficient to serve as a driving mutation to initiate a cascade of changes in mRNA and miRNA whose biological functions are important for maintaining proper chromosome number and structure and proliferative control (Tables 1 and 2). We show that these changes translate into biological effects, since cells expressing PAX3-FOXO1 are primarily aneuploid (Table 3A), have altered chromosome structure (Table 3B), and have compensated to override the anti-proliferative responses to aneuploidy in physiologically relevant primary myoblasts (Figure 2). Consistent with our previous work [26], we demonstrate that inhibiting PAX3-FOXO1 phosphorylation reverts many of the gene expression changes to background levels (Figure 3), which translate into biological effects by altering the extent of chromosome structural abnormalities (Figure 1 and Table 3B) and enhanced proliferation rates (Figure 2). Given that these changes result explicitly from the expression and phosphorylation of PAX3-FOXO1, we conclude that the acquisition of the oncogene is the initiating driver mutation in the development of ARMS.

Because the aneuploid state is detrimental to a cell, growth retardation is one of the first cellular responses [42, 43]. Therefore, in order for muscle cells to develop into ARMS, the cells containing the fusion protein must overcome this proliferative defect. Consistent with the cell's normal response to the aneuploid state, we saw changes in cell cycle regulatory genes that would be expected to inhibit cellular proliferation. However, we also found that the presence of the fusion protein *enhances* proliferation, which seems in direct contrast to altered cell cycle regulatory genes. This apparent contradiction can be explained by the fact that we also observed significant increases in the expression of growth factor related genes and proliferative transcription factors, several of which are known direct targets of PAX3-FOXO1 [20, 44]. Taken together, these results support the idea that the oncogenic fusion protein serves a second role as a driving mutation: it promotes the increased expression of growth factor related genes, either directly or indirectly through downstream effects, that are capable of negating the inhibitory changes in cell cycle regulatory proteins to override the growth retarding effects of the aneuploid state.

**Figure 4:**
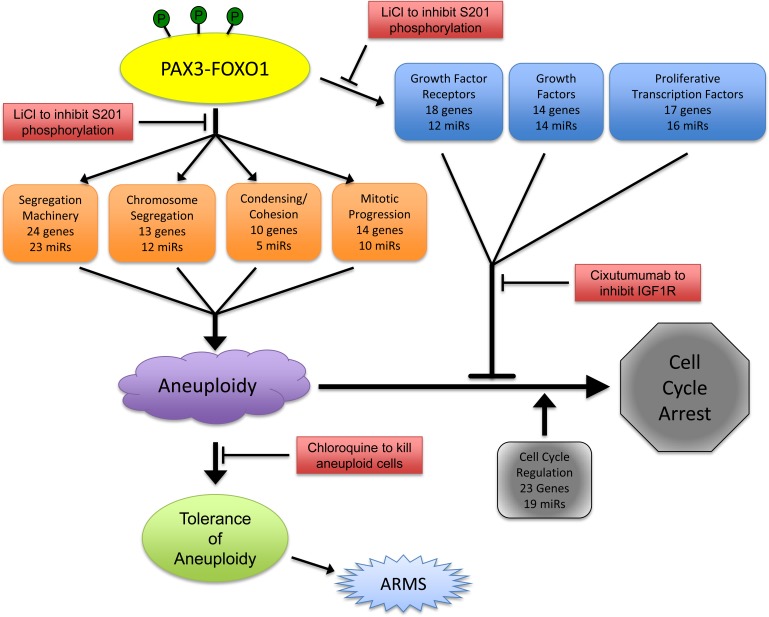
Model describing the role of PAX3-FOXO1 in the development of ARMS and how it informs potential therapy development

Based on the evidence presented here, we propose the following model by which PAX3-FOXO1 serves as the initial driving mutation in the development of ARMS (Figure 4). In this model myogenic cells in situ randomly and somatically acquire the t(2;13)(q35;q14) translocation, an event which is mimicked in our *in vitro* model system through the stable transduction of primary myoblasts. The subsequent expression of PAX3-FOXO1, maintained over repeated cellular duplications either in situ or *in vitro*, will bind to select promoters and regulatory sequences, thereby directly altering the expression of a subset of genes (Table 1, carats). These alterations could then initiate a cascade of indirect downstream events resulting in a global alteration of gene regulatory networks that ultimately reprogram proliferating myoblasts. This reprogramming results in the disruption of multiple aspects that lead to aneuploidy, including maintenance of the segregation machinery, regulation of chromosome segregation, promotion of chromosome cohesion and condensation, and insuring proper progression through mitosis.

In response to the aneuploid state, the cells attempt to halt proliferation, which is evidenced through our observed changes in the expression of cell cycle regulatory genes and miRNAs, and which most likely result from the aneuploid state and not from direct involvement of the fusion protein. However, PAX3-FOXO1 directly induces the expression of multiple and redundant proliferative genes, including growth factors, growth factor receptors, and proliferative transcription factors (Table 2, carats), thereby overriding aneuploidy-induced proliferation defects. Finally, the cells must be able to acquire a tolerance to the aneuploid state, most likely by affecting genes important in p53 regulation, in order to promote ARMS progression.

We reported that PAX3-FOXO1 is phosphorylated at Ser201 by GSK3β and small molecule inhibitors of this enzyme not only reduce phosphorylation at this site but also attenuate ARMS tumor phenotypes [26]. Consistent with this work, and adding to our present model, we found that phosphorylation contributes to the PAX3-FOXO1-dependent changes in gene and miRNA expression leading to the acquisition and persistence of aneuploidy. We found that although inhibiting phosphorylation of PAX3-FOXO1 at Ser201 and Ser205 did not affect aneuploidy, it altered the severity of chromosomal structural abnormalities (Figure 1 and Table 3B) and blocked the enhanced proliferation of primary myoblasts (Figure 2). We found phosphorylation contributes to the altered expression of multiple genes and miRNAs, which was evidenced by the reversion of gene expression to levels equivalent to the negative control in cells stably expressing phospho-incompetent mutants (Figure 3). Therefore, our results provide additional evidence to support the role post-translational modifications make in regulating the contributions of an oncogene to the development of a solid tumor. Further, these results identify this event, the specific phosphorylation of PAX3-FOXO1, as a viable biological target for therapy development (Figure 4). Interestingly, inhibition of phosphorylation at Ser209 enhanced the severity of sister chromatid dissociation (Figure 1D and Table 3B), and had proliferation rates indistinguishable from the negative control (Figure 1). Although we previously reported undetectable levels of phosphorylation of PAX3-FOXO1 at Ser209 in this system [28], the results presented here would suggest that this event does occur at greatly reduced levels and below the limits of detection.

The results presented here are important for directing the development of new therapies for the treatment of ARMS (Figure 4) and provide implications for how others may approach the development of therapies for solid tumors. Many present experimental therapies for ARMS, and other solid tumors, focus on inhibiting a single gene or a single pathway genetically located downstream of the oncogene. However, these therapies have not proven effective in Phase I or Phase II clinical trials for ARMS [45–48]. These outcomes are not surprising given the global nature of mRNA and miRNA alterations seen in cells that express PAX3-FOXO1. As such, therapies targeting a single gene product or pathway would be expected to have limited efficacy since multiple genes with similar biological functions could easily compensate.

Therefore, we propose the development of a multi-faceted regimen that targets genetic or biological characteristics identified from our work. We published that small molecules such as LiCl attenuate ARMS tumor phenotypes [26]. Others demonstrated that compounds that interfere with growth factor dependent proliferation, such as Cixutumumab [48], or the survival of aneuploid cells, such as chloroquine [49], might be effective as new treatments for human solid tumors. Therefore, we propose a combination therapy that minimally utilizes these three drugs for the treatment of ARMS. This regimen is expected to target both the driver mutation, with LiCl or other GSK3beta inhibitors preventing the phosphorylation of PAX3-FOXO1 at Ser201 [26] thereby inhibiting altered gene expression and the biological consequences of this driver mutation, with chloroquine killing aneuploid cells while Cixutumumab removes the growth-factor-dependent proliferative advantage. This regimen is expected to be specific for ARMS tumor cells, since all targets derive from the presence of the unique genetic mutation, have minimal negative effects since all proposed drugs are proven safe in human subjects, and would inhibit three molecular events essential for the viability of ARMS tumor cells.

Finally, the paradigm created here has implications that may be applied to other tumor models. Studies are being published with more frequency that utilize deep-sequencing strategies to examine global gene and miRNA expression changes resulting from the presence of an oncogene or the process of malignant transformation for examples see [50–53]. Often a single gene, miRNA, or miRNA locus is selected from these global studies for more in depth investigation to utilize as a specific target for therapy development. However, given that our results demonstrate that individual tumor phenotypes (e.g., induction of aneuploidy) arise from alterations in multiple biological aspects of this phenotype (e.g., mitotic progression, segregation machinery, etc.), it would be expected that a cell would be capable of compensating for the therapeutic inhibition of a single gene or miRNA. Therefore, as illustrated in the present study, deep sequencing studies should be utilized to discover how global gene regulatory alterations affect biological phenotypes and then use this knowledge to develop a multi-faceted therapeutic approach to target specific aspects of multiple different tumor phenotypes that are essential for the progression of the disease.

## MATERIALS AND METHODS

### Primary cells and culture conditions

Mouse primary myoblasts were isolated from 2 - 4 day old C57/Bl6 mice as previously described [40]. Cells were grown as previously described [25–28, 40] and were passage-matched to prevent possible differences due to passage conditions.

### Stable transduction of primary myoblasts

Mouse primary myoblasts were stably transduced as previously described [24, 40] with the MSCV-IRES-puromycin empty vector, vector containing FLAG epitope-tagged Pax3 (FLAG-Pax3), FLAG-PAX3-FOXO1, or FLAG-PAX3-FOXO1 in which each of the previously identified phosphorylation sites [27, 28] were mutated to the phospho-incompetent alanine (S201A, S205A, or S209A) [27, 28]. Three days post-transduction, cells were selected using puromycin, as previously described [28]. The stably transduced cells were harvested and pooled from three independent transductions to create a single population that express each construct.

### Western blot analysis

Stably transduced cells were grown to 80% confluency, harvested, and total cell extracts made, as previously described [26–28, 40]. Equal amounts of total cell lysates (12μg) were separated by 8% SDS-PAGE and analyzed by Western blot analysis using antibodies specific for Pax3 [54] as previously described [27, 28].

### Metaphase chromosome analysis

Transduced primary myoblasts were grown until logarithmic phase growth (approximately 70 - 80% confluency). The cells were then treated with Colcemeid (100ng/ml), harvested, and prepared for metaphase chromosome analysis, as previously described [55]. Metaphase images were captured using an Applied Imaging Model ER-3339 cooled CCD camera (Applied Spectral Imaging) mounted on top of a Nikon Eclipse E400 with CytoVision version 3.1 image-capture software (Applied Spectral Imaging).

### RNA extraction, cDNA library construction, and cDNA deep sequencing

Primary myoblasts stably expressing empty vector, FLAG-PAX3, or FLAG-PAX3-FOXO1 were grown to approximately 80% confluency. Total RNA was isolated using the miRNeasy mini kit (Qiagen), allowing for the isolation of RNA <30 bp in length, according to the manufacturer's specifications. Poly-A^+^ mRNA or miRNA were isolated from 4μg total RNA, to generate the respective cDNA libraries, both using the Illumina sample preparation kits according to the manufacturer's specifications. The cDNA libraries were provided a unique index identifier, allowing the clustering of several samples into a single sequencing lane, and deep sequencing analyses were performed in triplicate from three independent cell growth, RNA isolation, and cDNA library constructions.

### mRNA-seq data analysis

The raw data was groomed and trimmed for quality of read using online Galaxy analysis (https://usegalaxy.org), resulting in 40 - 41 high quality base pair reads for each sequence with between 4 - 6 million independent reads for each sample. The sequences were mapped to the mouse genome using Tophat analysis, transcripts were assembled using the Cufflinks program, and individual replicates were merged into a single file using Cuffmerge. The resulting transcript reads were normalized using Fragments Per Kilobase of transcript per Million mapped reads (FPKM) analysis, which normalizes each identified sequence for the length of the identified transcript and the volume of the total read yield from each run. Differential expression was determined from these normalized values using the Cuffdiff program, which not only compares differential expression of the merged files between sets but also utilizes the sequence results from the three independent determinations within each set to assign statistical significance to the differential expression.

### miRNA-seq data analysis

Raw fastq sequences were obtained from the Illumina Genome Analyzer II using the “Demultiplex” algorithm in the CASAVA 1.8.2 software (Illumina) that allows the identification of individual samples by “index sequences” contained within the adapters and introduced during the adapter ligation and amplification of the samples. miRNAKey, a software package used for analyzing small RNA data obtained from deep sequencing experiments, was used for data analysis at default settings. The pipeline clips the Illumina 3′ adaptor sequences from the reads, maps the clipped reads to miRBase and uses the Seq-EM algorithm to estimate the distribution of multiply mapped reads across the observed miRNAs. Sequences less than 16 bases after adaptor clipping were removed. The read counts obtained were then used for differential expression analysis between control and experimental samples using EBSeq from the R package with a False Discovery Rate (FDR) of 5%. We used the default ‘Median Normalization’ in EBSeq to make the counts comparable across samples.

### Proliferation assay

The proliferation rate of cells stably expressing empty vector, FLAG-PAX3, FLAG-PAX3-FOXO1, or the phospho-incompetent mutants was assessed using the CCK-8 colorimetric assay kit, according to the manufacturer's specifications (Cell Counting Kit-8, Dojindo Molecular Technologies) and as previously described [26].

### qRT-PCR

Total RNA was extracted from cells stably expressing empty vector, FLAG-PAX3, FLAG-PAX3-FOXO1, or the FLAG-PAX3-FOXO1 phospho-mutants. Equal amounts of isolated RNA (100ng) were used for cDNA synthesis using the iScript cDNA synthesis kit (Bio-Rad) for mRNA and the Taqman miRNA reverse transcription kit (Applied Biosystems) for miRNA. The qRT-PCR was performed on the resulting cDNA using the CFX96 Touch™ Real-Time PCR Detection System using commercially available primer/probe sets and the Applied Biosystems Universal Master Mix, according to the manufacturer's specifications. All results were normalized for GAPDH (mRNA) or the U6 small nuclear RNA for miRNA and reported as fold expression relative to the results obtained for cells stably transduced with the empty vector.

## SUPPLEMENTARY FIGURES AND TABLES


