# Supplementary material for: Acquisition of an oncogenic fusion protein serves as an initial driving mutation by inducing aneuploidy and overriding proliferative defects
Source: Oncotarget. 2016 Aug 30;7(39):62814–35. doi: 10.18632/oncotarget.11716 (PMC5325330; doi:10.18632/oncotarget.11716)

Acquisition of an oncogenic fusion protein serves as an initial driving mutation by inducing aneuploidy and overriding proliferative defects

Supplementary Material

Supplementary Figure 1

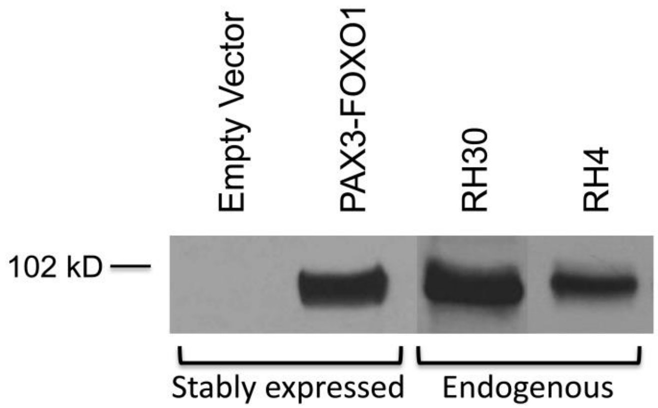

Supplement: Supplementary file 1 [file oncotarget-07-62814-s001.pdf]
